# Crystal structure of dimethyl 3,3′-[(4-chloro­phen­yl)methyl­ene]bis­(1*H*-indole-2-carboxyl­ate)

**DOI:** 10.1107/S1600536814020686

**Published:** 2014-09-27

**Authors:** Yu-long Li, Hong-shun Sun, Hong Jiang, Ning Xu, Hong Xu

**Affiliations:** a Chemical Engineering Department, Nanjing College of Chemical Technology, Geguan Road No.265 Nanjing, Nanjing 210048, People’s Republic of China

**Keywords:** crystal structure, indole, bis-indolymethane, MRI contrast agent, N—H⋯O hydrogen bonds, C—H⋯π inter­actions

## Abstract

In the title compound, C_27_H_21_ClN_2_O_4_, the mean planes of the two indole ring systems (r.m.s. deviations = 0.021 and 0.024 Å) are approximately perpendicular to one another, with a dihedral angle of 79.54 (12)°. The benzene ring is twisted with respect to the mean planes of the two indole ring systems at angles of 80.14 (15) and 86.30 (15)°. In the crystal, mol­ecules are linked by N—H⋯O hydrogen bonds, forming inversion dimers with an *R* ^2^_2_(18) ring motif. The dimers are linked by a further N—H⋯O hydrogen bond, forming chains along [100]. There are intra- and inter­molecular C—H⋯π inter­actions present, the latter linking the chains to form a three-dimensional supra­molecular structure.

## Abstract 2

In the crystal, mol­ecules are linked by N—H⋯O hydrogen bonds, forming inversion dimers, which are linked by a further N—H⋯O hydrogen bond, forming chains along [100]. There are intra- and inter­molecular C—H⋯π inter­actions present, the latter linking the chains to form a three-dimensional supra­molecular structure.

## Chemical context   

Indole derivatives are found abundantly in a variety of natural plants and exhibit various physiological properties (Poter *et al.*, 1977 ▶; Sundberg, 1996 ▶). Among them, bis-indolymethane derivatives have been found to be potentially bioactive compounds (Chang *et al.*, 1999 ▶; Ge *et al.*, 1999 ▶). In recent years, the synthesis and applications of bis-indolymethane derivatives have been studied widely. The title compound is one of the bis-indolymethane derivatives used as a precursor for MRI contrast agents (Ni, 2008 ▶). We report herein on its synthesis and crystal structure. Similar structures are reported by Sun *et al.* (2012 ▶, 2013 ▶).
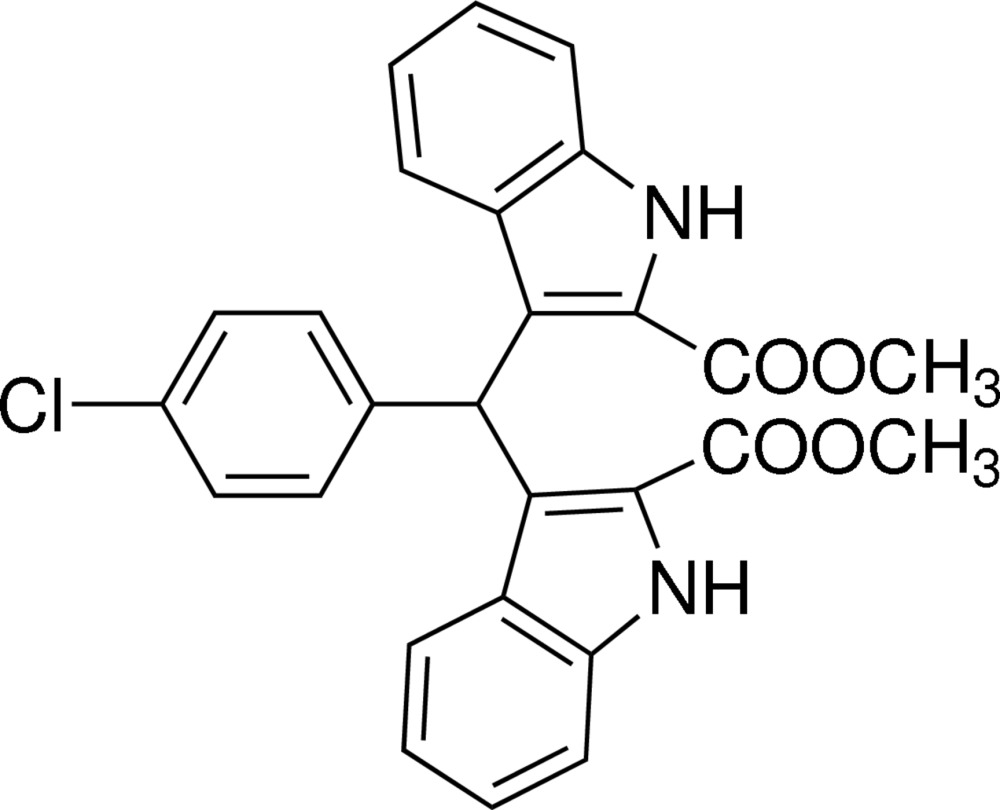


## Structural commentary   

**Figure 1:**
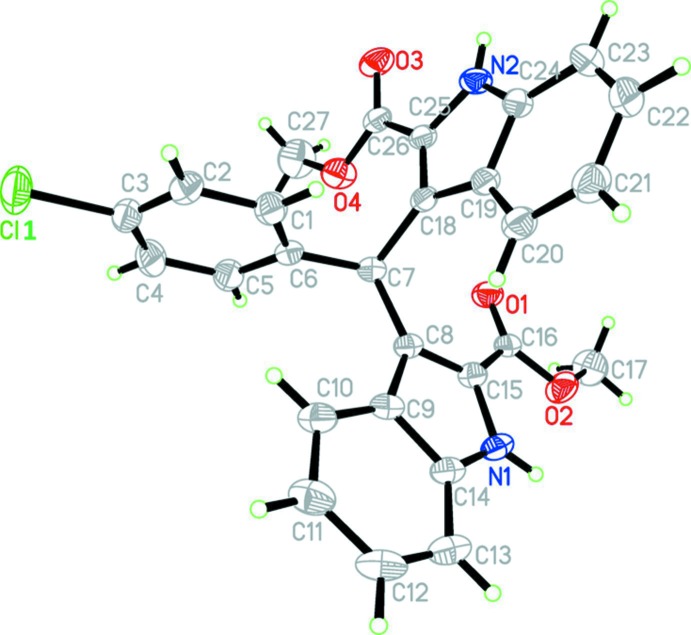
The mol­ecular structure of the title mol­ecule, with the atom-labelling scheme. Displacement ellipsoids are drawn at the 30% probability level.

The mol­ecular structure of the title compound is shown in Fig. 1 ▶. The benzene ring (C1–C6) is twisted with respect to the two indole rings, (N1/C8–C15) and (N2/CC18–C25), making dihedral angles of 80.14 (15) and 83.30 (15)°, respectively. The indole ring systems make a dihedral angle of 79.54 (12)°.

**Figure 2:**
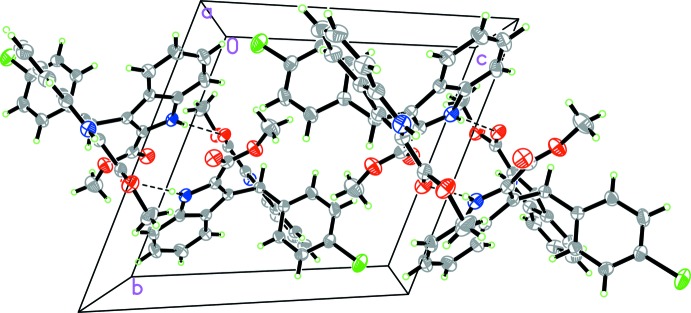
A perspective view along the *a* axis of the crystal packing of the title compound. Hydrogen bonds are shown as dashed lines (see Table 1 ▶ for details).

In the crystal, mol­ecules are linked by N—H⋯O hydrogen bonds, forming inversion dimers with an 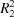
(18) ring motif (Fig. 2 ▶ and Table 1 ▶). The dimers are linked by a further N—H⋯O hydrogen bond, forming chains along [100] (Fig. 2 ▶ and Table 1 ▶). There are intra- and inter­molecular C—H⋯π inter­actions present (Table 1 ▶); the latter link the chains to form a three-dimensional supra­molecular structure.

## Synthesis and crystallization   

Methyl indole-2-carboxyl­ate (17.5 g, 100 mmol) was dissolved in 200 ml methanol; 4-chloro­benzaldehyde (7.0 g, 50 mmol) was added and the mixture heated to reflux. Concentrated HCl (3.7 ml) was added and the reaction was left for 1 h. After cooling the white product formed was filtered off and washed thoroughly with methanol; yield 95%. The reaction was followed by TLC (CHCl_3_:hexane = 1:1). Crystals of the title compound suitable for X-ray analysis were obtained by slow evaporation of an ethanol solution.

## Refinement   

Crystal data, data collection and structure refinement details are summarized in Table 2 ▶. H atoms were positioned geometrically, and constrained to ride on their parent atoms: N—H = 0.86 Å and C—H = 0.93, 0.96, and 0.98 Å for aromatic, methyl and methine H atoms, respectively, with *U*_iso_(H) = 1.5U_eq_(C) for methyl H atoms and = 1.2U_eq_(N,C) for other H atoms.

## Supplementary Material

Crystal structure: contains datablock(s) I, New_Global_Publ_Block. DOI: 10.1107/S1600536814020686/su2784sup1.cif

Structure factors: contains datablock(s) I. DOI: 10.1107/S1600536814020686/su2784Isup2.hkl

Supporting information file. DOI: 10.1107/S1600536814020686/su2784Isup3.cml

CCDC reference: 1024391

Additional supporting information:  crystallographic information; 3D view; checkCIF report

## Figures and Tables

**Table 1:** Hydrogen-bond geometry (Å, °) *Cg*1, *Cg*3, *Cg*4 and *Cg*5 are the centroids of the N1/C8/C9/C14/C15, C1–C6, C9–C14 and C19–C24 rings, respectively.

*D*—H⋯*A*	*D*—H	H⋯*A*	*D*⋯*A*	*D*—H⋯*A*
N2—H2*A*⋯O1^i^	0.86	2.04	2.862 (3)	159
N1—H1*A*⋯O3^ii^	0.86	2.08	2.923 (4)	168
C20—H20*A*⋯*Cg*1	0.93	2.89	3.568 (4)	131
C10—H10*A*⋯*Cg*3	0.93	2.90	3.705 (5)	146
C27—H27*A*⋯*Cg*4^iii^	0.96	2.78	3.719 (5)	166
C11—H11*A*⋯*Cg*5^iv^	0.93	2.88	3.750 (4)	156

Symmetry codes: (i) 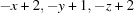; (ii) 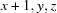; (iii) 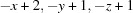; (iv) 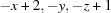.

**Table 2:** Experimental details

Crystal data	
Chemical formula	C_27_H_21_ClN_2_O_4_
*M*_r_	472.91
Crystal system, space group	Triclinic, *P* 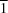
Temperature (K)	293
*a*, *b*, *c* (Å)	10.126 (2), 11.090 (2), 12.246 (2)
α, β, γ (°)	109.58 (3), 111.50 (3), 91.32 (3)
*V* (Å^3^)	1188.7 (4)
*Z*	2
Radiation type	Mo *K*α
μ (mm^−1^)	0.20
Crystal size (mm)	0.30 × 0.20 × 0.10

Data collection	
Diffractometer	Enraf–Nonius CAD-4
Absorption correction	ψ scan (North *et al.*, 1968 ▶)
*T*_min_, *T*_max_	0.943, 0.981
No. of measured, independent and observed [*I* > 2σ(*I*)] reflections	4651, 4381, 2728
*R*_int_	0.024
(sin θ/λ)_max_ (Å^−1^)	0.603

Refinement	
*R*[*F* ^2^ > 2σ(*F* ^2^)], *wR*(*F* ^2^), *S*	0.057, 0.177, 1.01
No. of reflections	4381
No. of parameters	307
H-atom treatment	H-atom parameters constrained
Δρ_max_, Δρ_min_ (e Å^−3^)	0.18, −0.33

Computer programs: *CAD-4 EXPRESS* (Enraf–Nonius, 1994 ▶), *XCAD4* (Harms & Wocadlo, 1995 ▶), and *SHELXS97*, *SHELXL97* and *SHELXTL* (Sheldrick, 2008 ▶).

## supplementary crystallographic information

### Crystal data

**Table:**

C_27_H_21_ClN_2_O_4_	*Z* = 2
*M**_r_* = 472.91	*F*(000) = 492
Triclinic, *P*1	*D*_x_ = 1.321 Mg m^−^^3^
Hall symbol: -P 1	Mo *K*α radiation, λ = 0.71073 Å
*a* = 10.126 (2) Å	Cell parameters from 25 reflections
*b* = 11.090 (2) Å	θ = 9–13°
*c* = 12.246 (2) Å	µ = 0.20 mm^−^^1^
α = 109.58 (3)°	*T* = 293 K
β = 111.50 (3)°	Block, colourless
γ = 91.32 (3)°	0.30 × 0.20 × 0.10 mm
*V* = 1188.7 (4) Å^3^	

### Data collection

**Table:**

Enraf–Nonius CAD-4 diffractometer	2728 reflections with *I* > 2σ(*I*)
Radiation source: fine-focus sealed tube	*R*_int_ = 0.024
Graphite monochromator	θ_max_ = 25.4°, θ_min_ = 1.9°
ω/2θ scans	*h* = 0→12
Absorption correction: ψ scan (North *et al.*, 1968)	*k* = −13→13
*T*_min_ = 0.943, *T*_max_ = 0.981	*l* = −14→13
4651 measured reflections	3 standard reflections every 200 reflections
4381 independent reflections	intensity decay: 1%

### Refinement

**Table:**

Refinement on *F*^2^	Primary atom site location: structure-invariant direct methods
Least-squares matrix: full	Secondary atom site location: difference Fourier map
*R*[*F*^2^ > 2σ(*F*^2^)] = 0.057	Hydrogen site location: inferred from neighbouring sites
*wR*(*F*^2^) = 0.177	H-atom parameters constrained
*S* = 1.01	*w* = 1/[σ^2^(*F*_o_^2^) + (0.1*P*)^2^ + 0.1*P*] where *P* = (*F*_o_^2^ + 2*F*_c_^2^)/3
4381 reflections	(Δ/σ)_max_ < 0.001
307 parameters	Δρ_max_ = 0.18 e Å^−^^3^
0 restraints	Δρ_min_ = −0.33 e Å^−^^3^

### Special details

**Table:**

Geometry. All e.s.d.'s (except the e.s.d. in the dihedral angle between two l.s. planes) are estimated using the full covariance matrix. The cell e.s.d.'s are taken into account individually in the estimation of e.s.d.'s in distances, angles and torsion angles; correlations between e.s.d.'s in cell parameters are only used when they are defined by crystal symmetry. An approximate (isotropic) treatment of cell e.s.d.'s is used for estimating e.s.d.'s involving l.s. planes.
Refinement. Refinement of *F*^2^ against ALL reflections. The weighted *R*-factor *wR* and goodness of fit *S* are based on *F*^2^, conventional *R*-factors *R* are based on *F*, with *F* set to zero for negative *F*^2^. The threshold expression of *F*^2^ > σ(*F*^2^) is used only for calculating *R*-factors(gt) *etc*. and is not relevant to the choice of reflections for refinement. *R*-factors based on *F*^2^ are statistically about twice as large as those based on *F*, and *R*- factors based on ALL data will be even larger.

### Fractional atomic coordinates and isotropic or equivalent isotropic displacement parameters (Å^2^)

**Table:**

	*x*	*y*	*z*	*U*_iso_*/*U*_eq_	
Cl1	0.48461 (11)	0.07229 (11)	0.16877 (9)	0.0792 (4)	
O1	1.1884 (2)	0.5955 (2)	0.8803 (2)	0.0542 (6)	
N1	1.3863 (3)	0.3971 (3)	0.7210 (3)	0.0494 (7)	
H1A	1.4745	0.4332	0.7676	0.059*	
C1	0.8128 (3)	0.1650 (3)	0.5022 (3)	0.0481 (8)	
H1B	0.8548	0.1250	0.5593	0.058*	
N2	0.8964 (3)	0.3420 (3)	0.9111 (2)	0.0472 (7)	
H2A	0.8496	0.3612	0.9590	0.057*	
O2	1.4243 (2)	0.6008 (2)	0.9253 (2)	0.0643 (7)	
C2	0.6959 (3)	0.0963 (3)	0.3918 (3)	0.0486 (8)	
H2B	0.6599	0.0110	0.3745	0.058*	
O3	0.6953 (2)	0.4961 (2)	0.8462 (2)	0.0649 (7)	
C3	0.6339 (3)	0.1556 (3)	0.3084 (3)	0.0505 (8)	
O4	0.7917 (3)	0.5349 (2)	0.7210 (2)	0.0649 (7)	
C4	0.6861 (4)	0.2805 (4)	0.3326 (3)	0.0636 (10)	
H4A	0.6429	0.3198	0.2752	0.076*	
C5	0.8042 (4)	0.3484 (3)	0.4436 (3)	0.0544 (9)	
H5A	0.8401	0.4334	0.4599	0.065*	
C6	0.8694 (3)	0.2916 (3)	0.5305 (3)	0.0392 (7)	
C7	0.9952 (3)	0.3692 (3)	0.6546 (3)	0.0379 (7)	
H7A	0.9867	0.4608	0.6708	0.045*	
C8	1.1433 (3)	0.3553 (3)	0.6522 (3)	0.0387 (7)	
C9	1.1884 (3)	0.2641 (3)	0.5618 (3)	0.0420 (7)	
C10	1.1168 (4)	0.1620 (3)	0.4438 (3)	0.0520 (8)	
H10A	1.0169	0.1414	0.4082	0.062*	
C11	1.1962 (4)	0.0937 (3)	0.3823 (4)	0.0610 (10)	
H11A	1.1492	0.0269	0.3042	0.073*	
C12	1.3474 (4)	0.1226 (4)	0.4348 (4)	0.0628 (10)	
H12A	1.3983	0.0729	0.3917	0.075*	
C13	1.4211 (4)	0.2216 (3)	0.5474 (4)	0.0586 (9)	
H13A	1.5211	0.2412	0.5814	0.070*	
C14	1.3401 (3)	0.2928 (3)	0.6098 (3)	0.0458 (8)	
C15	1.2682 (3)	0.4352 (3)	0.7465 (3)	0.0421 (7)	
C16	1.2854 (3)	0.5492 (3)	0.8566 (3)	0.0435 (7)	
C17	1.4544 (5)	0.7217 (4)	1.0310 (4)	0.0861 (14)	
H17A	1.5566	0.7502	1.0740	0.129*	
H17B	1.4165	0.7089	1.0883	0.129*	
H17C	1.4102	0.7863	1.0007	0.129*	
C18	0.9788 (3)	0.3408 (3)	0.7625 (3)	0.0377 (7)	
C19	1.0442 (3)	0.2506 (3)	0.8189 (3)	0.0407 (7)	
C20	1.1385 (3)	0.1625 (3)	0.7983 (3)	0.0501 (8)	
H20A	1.1738	0.1553	0.7366	0.060*	
C21	1.1774 (4)	0.0877 (4)	0.8703 (4)	0.0604 (9)	
H21A	1.2390	0.0289	0.8567	0.073*	
C22	1.1255 (4)	0.0985 (4)	0.9646 (4)	0.0640 (10)	
H22A	1.1550	0.0474	1.0131	0.077*	
C23	1.0335 (4)	0.1817 (3)	0.9869 (3)	0.0565 (9)	
H23A	1.0004	0.1889	1.0500	0.068*	
C24	0.9908 (3)	0.2555 (3)	0.9116 (3)	0.0447 (8)	
C25	0.8887 (3)	0.3933 (3)	0.8212 (3)	0.0407 (7)	
C26	0.7834 (3)	0.4793 (3)	0.7995 (3)	0.0459 (8)	
C27	0.6854 (5)	0.6167 (4)	0.6893 (5)	0.0922 (15)	
H27A	0.7009	0.6511	0.6319	0.138*	
H27B	0.6943	0.6868	0.7648	0.138*	
H27C	0.5907	0.5662	0.6501	0.138*	

### Atomic displacement parameters (Å^2^)

**Table:**

	*U*^11^	*U*^22^	*U*^33^	*U*^12^	*U*^13^	*U*^23^
Cl1	0.0580 (6)	0.0938 (8)	0.0525 (6)	0.0045 (5)	0.0014 (4)	0.0102 (5)
O1	0.0494 (14)	0.0606 (14)	0.0504 (13)	0.0031 (11)	0.0266 (11)	0.0107 (11)
N1	0.0327 (14)	0.0601 (17)	0.0575 (17)	0.0042 (12)	0.0215 (13)	0.0205 (14)
C1	0.0460 (18)	0.0504 (19)	0.0497 (18)	0.0086 (15)	0.0175 (15)	0.0222 (16)
N2	0.0437 (15)	0.0562 (16)	0.0462 (15)	0.0066 (13)	0.0277 (13)	0.0134 (13)
O2	0.0417 (13)	0.0720 (16)	0.0589 (14)	−0.0074 (12)	0.0128 (11)	0.0088 (13)
C2	0.0441 (18)	0.0450 (18)	0.0527 (19)	0.0006 (15)	0.0176 (16)	0.0154 (16)
O3	0.0427 (13)	0.0794 (18)	0.0800 (17)	0.0187 (12)	0.0360 (13)	0.0249 (14)
C3	0.0404 (18)	0.060 (2)	0.0435 (18)	0.0072 (16)	0.0130 (15)	0.0140 (16)
O4	0.0632 (16)	0.0732 (16)	0.0851 (18)	0.0372 (13)	0.0431 (14)	0.0447 (15)
C4	0.064 (2)	0.077 (3)	0.052 (2)	0.014 (2)	0.0130 (18)	0.038 (2)
C5	0.053 (2)	0.057 (2)	0.054 (2)	0.0014 (16)	0.0153 (17)	0.0287 (17)
C6	0.0328 (15)	0.0458 (17)	0.0434 (16)	0.0069 (13)	0.0193 (13)	0.0169 (14)
C7	0.0379 (16)	0.0390 (16)	0.0417 (16)	0.0073 (13)	0.0209 (13)	0.0150 (13)
C8	0.0359 (16)	0.0418 (16)	0.0437 (16)	0.0053 (13)	0.0202 (13)	0.0174 (14)
C9	0.0438 (17)	0.0433 (17)	0.0496 (18)	0.0077 (14)	0.0279 (15)	0.0195 (15)
C10	0.0517 (19)	0.0517 (19)	0.059 (2)	0.0041 (16)	0.0320 (17)	0.0169 (17)
C11	0.075 (3)	0.050 (2)	0.064 (2)	0.0047 (18)	0.043 (2)	0.0122 (18)
C12	0.073 (3)	0.055 (2)	0.082 (3)	0.0176 (19)	0.057 (2)	0.022 (2)
C13	0.050 (2)	0.063 (2)	0.080 (3)	0.0171 (17)	0.0406 (19)	0.030 (2)
C14	0.0425 (18)	0.0501 (19)	0.0564 (19)	0.0112 (15)	0.0290 (15)	0.0232 (16)
C15	0.0378 (16)	0.0469 (17)	0.0461 (17)	0.0055 (14)	0.0200 (14)	0.0190 (15)
C16	0.0374 (17)	0.0534 (19)	0.0427 (17)	−0.0011 (15)	0.0181 (14)	0.0194 (15)
C17	0.077 (3)	0.083 (3)	0.058 (2)	−0.031 (2)	0.012 (2)	−0.002 (2)
C18	0.0292 (14)	0.0415 (16)	0.0382 (15)	0.0022 (12)	0.0131 (12)	0.0104 (13)
C19	0.0333 (15)	0.0455 (17)	0.0398 (16)	0.0016 (13)	0.0139 (13)	0.0125 (14)
C20	0.0413 (18)	0.056 (2)	0.058 (2)	0.0120 (15)	0.0219 (16)	0.0249 (17)
C21	0.048 (2)	0.063 (2)	0.079 (3)	0.0170 (17)	0.0232 (19)	0.038 (2)
C22	0.055 (2)	0.076 (3)	0.068 (2)	0.009 (2)	0.0157 (19)	0.045 (2)
C23	0.055 (2)	0.072 (2)	0.0490 (19)	0.0034 (19)	0.0202 (17)	0.0316 (18)
C24	0.0423 (17)	0.0509 (19)	0.0394 (16)	0.0007 (15)	0.0160 (14)	0.0154 (15)
C25	0.0334 (15)	0.0467 (17)	0.0413 (16)	0.0015 (13)	0.0177 (13)	0.0125 (14)
C26	0.0348 (16)	0.0480 (18)	0.0482 (18)	0.0024 (14)	0.0178 (14)	0.0085 (15)
C27	0.088 (3)	0.090 (3)	0.117 (4)	0.050 (3)	0.043 (3)	0.056 (3)

### Geometric parameters (Å, º)

**Table:**

Cl1—C3	1.745 (3)	C9—C14	1.413 (4)
O1—C16	1.203 (4)	C10—C11	1.370 (5)
N1—C14	1.365 (4)	C10—H10A	0.9300
N1—C15	1.384 (4)	C11—C12	1.406 (5)
N1—H1A	0.8600	C11—H11A	0.9300
C1—C2	1.380 (4)	C12—C13	1.363 (5)
C1—C6	1.382 (4)	C12—H12A	0.9300
C1—H1B	0.9300	C13—C14	1.402 (4)
N2—C24	1.370 (4)	C13—H13A	0.9300
N2—C25	1.378 (4)	C15—C16	1.457 (4)
N2—H2A	0.8600	C17—H17A	0.9600
O2—C16	1.336 (4)	C17—H17B	0.9600
O2—C17	1.448 (4)	C17—H17C	0.9600
C2—C3	1.367 (5)	C18—C25	1.383 (4)
C2—H2B	0.9300	C18—C19	1.441 (4)
O3—C26	1.213 (4)	C19—C20	1.408 (4)
C3—C4	1.365 (5)	C19—C24	1.412 (4)
O4—C26	1.329 (4)	C20—C21	1.368 (5)
O4—C27	1.451 (4)	C20—H20A	0.9300
C4—C5	1.388 (5)	C21—C22	1.406 (5)
C4—H4A	0.9300	C21—H21A	0.9300
C5—C6	1.384 (4)	C22—C23	1.361 (5)
C5—H5A	0.9300	C22—H22A	0.9300
C6—C7	1.526 (4)	C23—C24	1.392 (5)
C7—C18	1.520 (4)	C23—H23A	0.9300
C7—C8	1.521 (4)	C25—C26	1.457 (4)
C7—H7A	0.9800	C27—H27A	0.9600
C8—C15	1.382 (4)	C27—H27B	0.9600
C8—C9	1.448 (4)	C27—H27C	0.9600
C9—C10	1.411 (4)		
			
C14—N1—C15	108.9 (3)	C14—C13—H13A	121.4
C14—N1—H1A	125.6	N1—C14—C13	129.0 (3)
C15—N1—H1A	125.6	N1—C14—C9	108.2 (3)
C2—C1—C6	122.0 (3)	C13—C14—C9	122.8 (3)
C2—C1—H1B	119.0	C8—C15—N1	110.2 (3)
C6—C1—H1B	119.0	C8—C15—C16	129.1 (3)
C24—N2—C25	108.9 (2)	N1—C15—C16	120.7 (3)
C24—N2—H2A	125.5	O1—C16—O2	123.8 (3)
C25—N2—H2A	125.5	O1—C16—C15	125.2 (3)
C16—O2—C17	116.1 (3)	O2—C16—C15	110.9 (3)
C3—C2—C1	118.9 (3)	O2—C17—H17A	109.5
C3—C2—H2B	120.5	O2—C17—H17B	109.5
C1—C2—H2B	120.5	H17A—C17—H17B	109.5
C4—C3—C2	121.1 (3)	O2—C17—H17C	109.5
C4—C3—Cl1	118.7 (3)	H17A—C17—H17C	109.5
C2—C3—Cl1	120.2 (3)	H17B—C17—H17C	109.5
C26—O4—C27	116.5 (3)	C25—C18—C19	106.3 (3)
C3—C4—C5	119.4 (3)	C25—C18—C7	125.4 (3)
C3—C4—H4A	120.3	C19—C18—C7	128.2 (2)
C5—C4—H4A	120.3	C20—C19—C24	118.2 (3)
C6—C5—C4	121.0 (3)	C20—C19—C18	135.0 (3)
C6—C5—H5A	119.5	C24—C19—C18	106.7 (3)
C4—C5—H5A	119.5	C21—C20—C19	119.1 (3)
C1—C6—C5	117.5 (3)	C21—C20—H20A	120.4
C1—C6—C7	121.9 (3)	C19—C20—H20A	120.4
C5—C6—C7	120.5 (3)	C20—C21—C22	121.0 (3)
C18—C7—C8	112.7 (2)	C20—C21—H21A	119.5
C18—C7—C6	110.0 (2)	C22—C21—H21A	119.5
C8—C7—C6	114.8 (2)	C23—C22—C21	121.8 (3)
C18—C7—H7A	106.2	C23—C22—H22A	119.1
C8—C7—H7A	106.2	C21—C22—H22A	119.1
C6—C7—H7A	106.2	C22—C23—C24	117.4 (3)
C15—C8—C9	105.6 (3)	C22—C23—H23A	121.3
C15—C8—C7	123.1 (3)	C24—C23—H23A	121.3
C9—C8—C7	131.3 (3)	N2—C24—C23	129.3 (3)
C10—C9—C14	117.8 (3)	N2—C24—C19	108.3 (3)
C10—C9—C8	135.1 (3)	C23—C24—C19	122.5 (3)
C14—C9—C8	107.1 (3)	N2—C25—C18	109.8 (3)
C11—C10—C9	119.3 (3)	N2—C25—C26	116.6 (3)
C11—C10—H10A	120.4	C18—C25—C26	133.4 (3)
C9—C10—H10A	120.4	O3—C26—O4	123.4 (3)
C10—C11—C12	121.3 (3)	O3—C26—C25	123.4 (3)
C10—C11—H11A	119.3	O4—C26—C25	113.2 (3)
C12—C11—H11A	119.3	O4—C27—H27A	109.5
C13—C12—C11	121.6 (3)	O4—C27—H27B	109.5
C13—C12—H12A	119.2	H27A—C27—H27B	109.5
C11—C12—H12A	119.2	O4—C27—H27C	109.5
C12—C13—C14	117.2 (3)	H27A—C27—H27C	109.5
C12—C13—H13A	121.4	H27B—C27—H27C	109.5
			
C6—C1—C2—C3	−0.4 (5)	C14—N1—C15—C16	−176.7 (3)
C1—C2—C3—C4	0.2 (5)	C17—O2—C16—O1	−2.2 (5)
C1—C2—C3—Cl1	−179.2 (2)	C17—O2—C16—C15	174.5 (3)
C2—C3—C4—C5	0.1 (5)	C8—C15—C16—O1	−2.9 (5)
Cl1—C3—C4—C5	179.6 (3)	N1—C15—C16—O1	173.6 (3)
C3—C4—C5—C6	−0.4 (6)	C8—C15—C16—O2	−179.6 (3)
C2—C1—C6—C5	0.1 (5)	N1—C15—C16—O2	−3.1 (4)
C2—C1—C6—C7	177.8 (3)	C8—C7—C18—C25	151.8 (3)
C4—C5—C6—C1	0.2 (5)	C6—C7—C18—C25	−78.6 (3)
C4—C5—C6—C7	−177.4 (3)	C8—C7—C18—C19	−32.3 (4)
C1—C6—C7—C18	−39.6 (4)	C6—C7—C18—C19	97.2 (3)
C5—C6—C7—C18	138.0 (3)	C25—C18—C19—C20	176.3 (3)
C1—C6—C7—C8	88.9 (3)	C7—C18—C19—C20	−0.2 (5)
C5—C6—C7—C8	−93.6 (3)	C25—C18—C19—C24	−1.0 (3)
C18—C7—C8—C15	−65.3 (4)	C7—C18—C19—C24	−177.5 (3)
C6—C7—C8—C15	167.7 (3)	C24—C19—C20—C21	−1.5 (4)
C18—C7—C8—C9	113.9 (3)	C18—C19—C20—C21	−178.6 (3)
C6—C7—C8—C9	−13.2 (4)	C19—C20—C21—C22	−0.5 (5)
C15—C8—C9—C10	−177.0 (3)	C20—C21—C22—C23	1.0 (6)
C7—C8—C9—C10	3.8 (6)	C21—C22—C23—C24	0.6 (5)
C15—C8—C9—C14	1.7 (3)	C25—N2—C24—C23	179.4 (3)
C7—C8—C9—C14	−177.5 (3)	C25—N2—C24—C19	−0.4 (3)
C14—C9—C10—C11	1.4 (5)	C22—C23—C24—N2	177.5 (3)
C8—C9—C10—C11	179.9 (3)	C22—C23—C24—C19	−2.8 (5)
C9—C10—C11—C12	0.5 (5)	C20—C19—C24—N2	−176.9 (3)
C10—C11—C12—C13	−1.7 (6)	C18—C19—C24—N2	0.9 (3)
C11—C12—C13—C14	0.8 (5)	C20—C19—C24—C23	3.3 (5)
C15—N1—C14—C13	−179.6 (3)	C18—C19—C24—C23	−178.9 (3)
C15—N1—C14—C9	0.7 (3)	C24—N2—C25—C18	−0.2 (3)
C12—C13—C14—N1	−178.5 (3)	C24—N2—C25—C26	174.8 (3)
C12—C13—C14—C9	1.2 (5)	C19—C18—C25—N2	0.8 (3)
C10—C9—C14—N1	177.5 (3)	C7—C18—C25—N2	177.4 (3)
C8—C9—C14—N1	−1.5 (3)	C19—C18—C25—C26	−173.1 (3)
C10—C9—C14—C13	−2.3 (5)	C7—C18—C25—C26	3.5 (5)
C8—C9—C14—C13	178.8 (3)	C27—O4—C26—O3	−1.7 (5)
C9—C8—C15—N1	−1.3 (3)	C27—O4—C26—C25	176.8 (3)
C7—C8—C15—N1	178.0 (3)	N2—C25—C26—O3	−7.7 (4)
C9—C8—C15—C16	175.5 (3)	C18—C25—C26—O3	165.9 (3)
C7—C8—C15—C16	−5.2 (5)	N2—C25—C26—O4	173.8 (3)
C14—N1—C15—C8	0.4 (3)	C18—C25—C26—O4	−12.6 (5)

### Hydrogen-bond geometry (Å, º)

Cg1, Cg3, Cg4 and Cg5 are the centroids of the N1/C8/C9/C14/C15, C1–C6, C9–C14 and C19–C24 rings, respectively.

**Table:**

*D*—H···*A*	*D*—H	H···*A*	*D*···*A*	*D*—H···*A*
N2—H2*A*···O1^i^	0.86	2.04	2.862 (3)	159
N1—H1*A*···O3^ii^	0.86	2.08	2.923 (4)	168
C20—H20*A*···*Cg*1	0.93	2.89	3.568 (4)	131
C10—H10*A*···*Cg*3	0.93	2.90	3.705 (5)	146
C27—H27*A*···*Cg*4^iii^	0.96	2.78	3.719 (5)	166
C11—H11*A*···*Cg*5^iv^	0.93	2.88	3.750 (4)	156

Symmetry codes: (i) −*x*+2, −*y*+1, −*z*+2; (ii) *x*+1, *y*, *z*; (iii) −*x*+2, −*y*+1, −*z*+1; (iv) −*x*+2, −*y*, −*z*+1.
